# Vitamin D receptor polymorphisms or serum levels as key drivers of breast cancer development? The question of the vitamin D pathway

**DOI:** 10.18632/oncotarget.14482

**Published:** 2017-01-04

**Authors:** Dino Amadori, Patrizia Serra, Nestory Masalu, Akwilina Pangan, Emanuela Scarpi, Aloyce Maria Bugingo, Deogratias Katabalo, Toni Ibrahim, Alberto Bongiovanni, Giacomo Miserocchi, Chiara Spadazzi, Chiara Liverani, Valentina Turri, Rosanna Tedaldi, Laura Mercatali

**Affiliations:** ^1^ Department of Medical Oncology, Istituto Scientifico Romagnolo per lo Studio e la Cura dei Tumori (IRST) IRCCS, Meldola, Italy; ^2^ Unit of Biostatistics and Clinical Trials, Istituto Scientifico Romagnolo per lo Studio e la Cura dei Tumori (IRST) IRCCS, Meldola, Italy; ^3^ Department of Oncology, Bugando Medical Center, Mwanza, Tanzania, Africa; ^4^ Osteoncology and Rare Tumors Center, Istituto Scientifico Romagnolo per lo Studio e la Cura dei Tumori (IRST) IRCCS, Meldola, Italy; ^5^ Healthcare Administration, Istituto Scientifico Romagnolo per lo Studio e la Cura dei Tumori (IRST) IRCCS, Meldola, Italy

**Keywords:** Africans, caucasians, 25(OH)D, vitamin D pathway, vitamin D binding protein

## Abstract

As total vitamin D levels are often lower in black than in white Americans, the former are frequently classified as vitamin D-deficient. To fully understand African vitamin D (25(OH)D) status, other factors should be considered, *e.g*. vitamin D blood carrier, vitamin D-binding protein (DBP), vitamin D receptor (*VDR*) and *DBP* polymorphisms. A prospective study on an indigenous black Tanzanian and a Caucasian Italian population was performed on 50 healthy donors from both populations and 35 Caucasian and 18 African breast cancer patients. 25(OH)D and DBP serum levels were analyzed by ELISA. A1012G, Cdx2 and Fok1 *VDR* polymorphisms and *DBP* polymorphisms rs4588 and rs7041 were genotyped by real-time PCR. Vitamin D and DBP levels were lower in healthy African donors than in Caucasians. Africans had a significantly higher frequency of AA and CC for Cdx2 and Fok1 polymorphisms, respectively. These allelic variants were related to a higher transcription of *VDR* gene and a higher activity of VDR receptor. With regard to polymorphism distribution, Africans showed innate higher levels and activity of VDR. We conclude that a strengthening of the vitamin D pathway could have a protective role against the development of breast cancer in the African population.

## INTRODUCTION

Ethnic differences play a role in circulating levels of serum vitamin D: black Americans are more likely to have lower levels of circulating 25(OH)D than white Americans, as darker skin pigmentation is associated with a slighter increase in serum concentration after a given amount of UVB exposure [1–5]. Vitamin D derived from diet and sunlight-driven synthesis in the skin is converted by the liver into its circulating form 25-hydroxyvitamin D (25(OH)D). 25(OH)D is thus transformed into the active form 1,25-dihydroxyvitamin D3 (calcitriol) in the kidney and in target tissues, including breast cancer (BC) cells [6]. Its classical physiological function is to regulate bone development and extracellular calcium concentration in the organism. The nonclassic functions of vitamin D range from hypertension and disturbed muscle function to susceptibility to infections autoimmune diseases and cancer. At a cellular level vitamin D cancer-related activity includes induction to cell differentiation, inhibition of proliferation by cell-cycle blockage or interference with signaling by growth factors, inducing apoptosis, stimulation of DNA damage repair, prevention of tumor angiogenesis, and inhibition of metastasis [7–9]. The observations of this spread of functions indicated a inhibitory role of vitamin D in cancer development.

In accordance with these data, vitamin D deficiency is common in patients diagnosed with BC, associating with worse prognosis and outcome [10]. In particular epidemiological data reported an inverse correlation between the risk of developing BC and serum 25(OH)D levels [11–13].

For these reasons the role of vitamin D supplementation in cancer prevention or in early-stage cancer patients has been investigated. Although most clinical data derive from studies showing a potential benefit of vitamin D supplementation in colon and breast cancer patients (BCPs), definitive randomized clinical trials of adequate size and duration with sufficient doses of vitamin D are still lacking [14–16].

Information on the vitamin D pathway activity is obtained not only through the observation of serum 25(OH)D levels. Vitamin D-binding protein (DBP) is the main blood carrier of vitamin D, binding around 85-90% of total circulating vitamin D. The vitamin-D fraction unbound to DBP is bound to albumin, with < 1% of total free vitamin D in blood [17].

According to the theory of the free hormones, vitamin D may enter target cells and work only when it is unbound to protein carriers [18–19]. DBP may thus be considered an important regulator of vitamin D activity. Inside the cell, most of the known effects of vitamin D are mediated via binding of the active forms of vitamin D to the vitamin D receptor (VDR), that is a regulator of the transcription expressed in almost all tissues, including normal breast and most BCs [20–22]. Common genetic polymorphisms in *VDR* and in *DBP* produce variant proteins which generate a different activity of the vitamin D pathway, especially in their affinity to vitamin D [23–24]. The prevalence of these polymorphisms differs among racial groups [8, 25–26]. Only Serum 25(OH)D levels have been evaluated in clinical practice [20, 25, 26], with a lack of information on the activation status of vitamin D.

Besides having lower levels of vitamin D, African Americans have a lower incidence of BC than Caucasians. The same trend has been observed in Native Africans. At the same time, as reported in our previous paper, [27] Tanzanian and Italian BCs present different histopathological and biomolecular characteristics: African patients have a higher frequency of negative prognostic markers, such as poor differentiation grade, ER-negative status, high proliferation index or advanced stage at diagnosis [28].

A comprehensive study on vitamin D pathway status and differences in African and Caucasian populations and their implication on BC is still missing. Moreover, most of the information available for Africans is gathered from studies on black Americans and only a few on native Africans [29–34].

We thus conducted a study in collaboration with the Bugando Medical Centre of Mwanza (Tanzania, Africa), to assess the levels of vitamin D and DBP and their relation to VDR and DBP protein genotypes between a native African and a Caucasian population, with the aim to study the differences in the vitamin D pathway between the two populations and their implications in cancer.

## RESULTS

### 25(OH)D and DBP circulating levels

#### Africans vs. Caucasians

Levels of 25(OH)D and DBP were significantly lower (*P* = 0.003 and *P* < 0.0001, respectively) in African than in Caucasian HDs, when considered as continuous variables (Table 1). In particular DBP was about threefold lower in African HDs (Table 1). DBP median levels were about threefold lower (*P* < 0.0001) also in African BCPs, with no change in 25(OH)D median levels.

**Table 1:** 25(OH)D and DBP in BCPs and HDs Median values (range) of 25(OH)D and DBP in BCPs and HDs

	Healthy			Patients		
	Africans (*n* = 50)	Caucasians (*n* = 50)	*P ^a^*	Africans (*n* = 18)	Caucasians (n = 35)	*P ^a^*
**25(OH)D(ng/ml)**	22.50 (5-94)	28.32 (11.26-60.70)	0.0030	33.50 (14-65)	27.01 (11.00-53.22)	0.1450
**DBP (μg/ml)**	75.13 (23.34-194.75)	219.62 (96.32-738.06)	<0.0001	87.89 (13.69–197.44)	227.98 (79.41–395.71)	<0.0001

^a^ median test.

Africans: BCPs vs. HDs: 25(OH)D: *P*^a^=0.013 DBP: *P*^a^=0.5150.

Caucasians: BCPs vs. HDs: 25(OH)D: P^a^=0.399 DBP: P^a^=0.5490.

A different distribution of vitamin D and DBP (*P* = 0.0073 and < 0.0001, respectively) was observed between African and Caucasian HDs, when considered as discrete variables with respect to the classes of vitamin D and DBP levels reported in the materials and methods section. In particular, a higher percentage of African HDs showed vitamin D levels < 20 ng/ml (38% *vs*. 8%, respectively). Similarly, most African HDs (64%) had low levels of DBP, compared to Caucasian HDs (0%, *P* < 0.001). These differences were lost when comparing BCPs (Table 2).

**Table 2:** 25(OH)D and DBP considered as discrete variables in BCPs and HDs

Marker	Africans		Caucasians	
	Healthy No. (%)	Patients No. (%)	Healthy No. (%)	Patients No. (%)
**25(OH)D (ng/ml)**				
** ≤ 20**	19 (38.0)	4 (22.2)	4 (8.0)	6 (17.1)
** 20.1-30**	19 (38.0)	4 (22.2)	27 (54.0)	16 (45.7)
** 30.1-50**	9 (18.0)	9 (50.0)	15 (30.0)	11 (31.1)
** > 50**	3 (6.0)	1 (5.6)	4 (8.0)	2 (5.7)
**DBP (μg/ml)**				
** ≤ 98**	32 (64.0)	9 (56.2)	0	2 (5.9)
** 98-198**	18 (36.0)	7 (43.8)	22 (47.8)	11 (32.3)
** > 198**	0	0	24 (52.2)	21 (61.8)

The Spearman's analyses revealed a direct correlation between 25(OH)D and DBP levels when considered as continuous variables. The increase in 25(OH)D was associated with an increase in DBP levels in the overall case series (r_s_=0.24; *P* = 0.003), in the Caucasian case series (r_s_=0.24; *P* = 0.018) and in the entire healthy subgroup (r_s_=0.31; *P* = 0.002).

#### HDs vs BCP within each population

Unlike DBP, vitamin D median levels were significantly higher in African BCPs than in HDs (Table 1) (*P* = 0.013). Level distribution of circulating markers was not significantly different between cases and controls, when considered as discrete variables (Table 2). Both markers showed no statistical difference in the Caucasian population.

#### SNP distribution

The genotype distributions of all selected polymorphisms from HDs and BCPs in both populations were analyzed by ethnicity for the Hardy-Weinberg Equilibrium. A1012G and Cdx2 polymorphisms were in linkage disequilibrium in both populations and therefore analyzed as haplotypes (three variants: Haplo1: CG, Haplo2: TG, and Haplo 3 TA) (Table 3). DBP haplotype analyses were performed only for Caucasian subjects, as the Africans reported no DBP polymorphisms in linkage disequilibrium (Haplo1: CG, Haplo2: CT, Haplo 3 AT and Haplo 4, others) (Table 3). We compared the allelic distributions of polymorphisms in our case series with those reported in European Caucasian and African populations extrapolated by the 1000 Genomes database (Table 4) [35]. We compared our Caucasian case series with an Italian Tuscany population, and our Tanzanian population with an American ancestry population (ASW) and an African population (YRI). The allelic frequency of all analyzed polymorphisms were homogeneous with the 1000 Genomes dataset for the Caucasian population. We observed a statistically significant difference in the African population for Cdx2 and rs4588, although our data showed a percentage trend similar to that reported by the 1000 Genome dataset (Table 4).

**Table 3:** Distribution of haplotypes in both populations

Haplotype	Africans		Caucasians	
	HealthyNo. (%)	PatientsNo. (%)	HealthyNo. (%)	PatientsNo (%)
**A1012A/Cdx2**				
**CG/CG**	1(2)	0 (0)	5 (10)	4 (11.4)
**CG/TG**	1 (2)	0 (0)	15 (30)	13 (37.1)
**CG/TA**	3 (6)	0 (0)	10 (20)	6 (17.1)
**TG/TG**	0 (0)	0 (0)	6 (12)	3 (8.6)
**TG/TA**	6 (12)	0 (0)	10 (20)	5 (14.3)
**TA/TA**	39 (78)	18 (100)	4 (8)	4 (11.4)
**rs7041/rs4588**				
**CG/CG**	-	-	16 (32)	12 (34.3)
**CT/CT**	-	-	1 (2)	1 (2.9)
**CT/other**	-	-	0 (0)	1 (2.9)
**AT/CG**	-	-	12 (24)	5 (14.3)
**AT/CT**	-	-	7 (14)	5 (14.3)
**AT/AT**	-	-	2 (4)	1 (2.86)
**AT/other**	-	-	12 (24)	10 (41.1)

**Table 4:** Comparison of allelic frequencies (%) of SNPs with those reported in the 1000 Genome Dataset

	A1012G				CDX2				FOK1				rs4588				rs7041			
		TSI*	C. S.	p		TSI	C. S.	p		TSI	C. S.	p		TSI	C. S.	p		TSI	C. S.	p
Caucasians	C	42	35		C	75	72		G	61	65		G	79	75		C	55	57	
	T	58	65	0.31	T	25	28	0.632	A	38	35	0.621	T	21	25	0.503	A	45	43	0.776
		AWS*				AWS				AWS				AWS				AWS		
Africans	C	12	6		C	28	13		G	80	84		G	78	91		C	16	8	
	T	88	94	0.139	T	72	87	**0.009**	A	20	16	0.463	T	22	8	**0.006**	A	84	92	0.082
		YRI*				YRI				YRI				YRI				YRI		
	C	1			C	2			G	81			G	96			C	3		
	T	99		0.118	T	98		**0.005**	A	19		0.578	T	3		0.122	A	96		0.126

* Data of allelic frequencies for the following populations: TSI, Tuscans in Italy; AWS, Americans of African Ancestry; YRI Yorubi in Nigeria.

C. S. Corrent series

### VDR SNPs

#### Africans vs Caucasians

African HDs showed a higher frequency of TT and AA homozygosity in A1012G and Cdx2 than Caucasian HDs (*P* < 0.0001 for both SNPs). Moreover, Africans had a higher CC frequency than Caucasians for Fok1 polymorphism (70% *vs*. 46%, respectively; *P* = 0.00253) (Table 5). We observed the same significant trends for A1012G and Cdx2 polymorphisms in BCPs: Africans showed 100% homozygosity for both SNPs (Table 5) compared to 34.3% and 11.4% observed in Caucasians. *Fok1*, instead, was similarly distributed in BCPs. The A1012G/Cdx2 haplotype was differently distributed among African and Caucasian HDs and BCPs (*P* < 0.0001 for both subgroups); In particular, 78% of African HDs and 100% of BCPs had haplotype TA/TA, compared to 8% and 14% of Caucasian HDs and BCPs, respectively (Table 3).

**Table 5:** Distribution of polymorphisms in both populations

	Healthy Donors			Patients		
	AfricansNo. (%)	CaucasiansNo. (%)	*P*	AfricansNo. (%)	CaucasiansNo. (%)	*P*
**A1012G**						
TT+CT	49 (98)	45 (90)		18 (100)	31 (88.6)	
CC	1 (2)	5 (10)	ns	0	4 (11.4)	ns
TT	45 (90)	20 (40)		18 (100)	12 (34.3)	
CT+CC	5 (10)	30 (60)	< 0.0001	0	23 (65.7)	< 0.0001
**Cdx2**						
AA+GA	48 (96)	24 (48)		18 (100)	15 (42.9)	
GG	2 (4)	26 (52)	< 0.0001	0	20 (57.1)	< 0.0001
AA	39 (78)	4 (8)		18 (100)	4 (11.4)	
GA+GG	11 (22)	46 (92)	< 0.0001	0	31 (88.6)	< 0.0001
**Fok1**						
TT+CT	15 (30)	27 (54)		11 (61.1)	21 (60)	
CC	35 (70)	23 (46)	0.00253	7 (38.9)	14 (40)	ns
TT	1 (2)	8 (16)		3 (16.7)	5 (14.3)	
CC+CT	49 (98)	42 (84)	0.03	15 (83.3)	30 (85.7)	ns
**rs4588**						
AA+CA	19 (38)	20 (40)		7 (38.6)	17 (48.6)	
CC	31 (62)	30 (60)	ns	11 (61.1)	18 (51.4)	ns
AA	0 (0)	1 (2)		0	2 (5.7)	
CA+CC	50 (100)	49 (98)	ns	18 (100)	33 (94.3)	ns
**rs7041**						
GG+TG	8 (16)	41 (82)		3 (16.7)	28 (80)	
TT	42 (84)	9 (18)	< 0.0001	15 (83.3)	7 (20)	< 0.0001
GG	0 (0)	16 (32)		0 (0)	13 (37.1)	
TG+TT	50 (100)	34 (68)	0.002	18 (100)	22 (66.0)	0.02

ns = not significant.

Comparison between cases and controls within each population.

The statistically significant comparisons in the African population were: Cdx2 AA vs. GA+GG (*P*=0.02); Fok1: TT+CTA vs. CC (*P*=0.02).

No statistically significant comparisons appeared within the Caucasian population.

#### HDs vs. BCP within each population

A1012G polymorphism was equally distributed in HDs and BCPs in both populations. African cases had a higher frequency of AA and TT homozygosity for Cdx2 and Fok1 SNPs, respectively (*P* = 0.02 in both cases). No significant difference was observed in the Caucasian population (Table 5). We observed no different distribution of haplotype A1012G/Cdx2 in either population (Table 3).

### DBP SNPs

#### Africans vs Caucasians

The rs4588 polymorphism was similarly distributed among African and Caucasian HDs and BCPs. C allele was the most frequent among all subgroups (Table 5). TT homozygotes for rs7041 were significantly more frequent in Africans HDs (84% *vs*. 18%, *P* < 0.0001). No African HD showed GG homozygosity. A similar distribution was observed in BCPs (Table 5).

#### HDs vs. BCP within each population

No difference was observed between cases and controls in either African or Caucasian population for rs4588 and rs7041 polymorphisms (Table 5). Haplotypes of rs7041/rs4588 polymorphisms were analyzed only in the Caucasian case series, since the two SNPs reported no Linkage disequilibrium in the African population. The distribution of this haplotype was not significantly different in either Caucasian BCPs or HDs (Table 3).

### 25(OH)D and DBP levels with respect to SNPs

#### VDR SNPs

We observed no difference in 25(OH)D and DBP levels with respect to the allelic variant of the *VDR* analyzed polymorphisms in both African HDs and BCPs (Table 6).

**Table 6:** 25(OH)D and DBP levels according to polymorphism distribution in the African population

	No.	Healthy Donors					Patients			
		25(OH)D (ng/ml)		DBP (μg/ml)		25(OH)D (ng/ml)			DBP (μg/ml)	
		Median value (range)	*P ^1^*	Median value (range)	*P ^1^*	No.	Median value (range)	*P ^1^*	Median value (range)	*P ^1^*
**A1012G**										
TT+CT	49	23 (5-94)		76.16 (31.53-194.75)		18	33.5 (14-65)		87.89 (13.69-197.44)	
CC	1	17	0.252	23.34	0.096	0	-	-	-	-
TT	45	23 (5-94)		74.11 (31.53-194.75)		18	33.5 (14-65)		87.89 (13.69-197.44)	
CT+CC	5	18 (17-66)	0.935	103.67 (23.34-119.45)	0.674	0	-	-	-	-
**Cdx2**										
AA+GA	48	23 (5-94)		75.13 (31.53-194.75)		18	33.5 (14-65)		87.89 (13.69-197.44)	
GG	2	17.5 (17-18)	0.150	63.50 (23.34-103.67)	0.569	0	-	-	-	-
AA	39	23 (9-53)		73.10 (31.53-194.75)		18	33.5 (14-65)		87.89 (13.69-197.44)	
GA+GG	11	18 (5-94)	0.742	103.67 (23.34-124.76)	0.454	0	-	-	-	-
**Fok1**										
TT+CT	15	23 (11-45)		73.10 (23.34-183.08)		11	30 (19-46)		85.29 (13.69-197.44)	
CC	35	22 (5-94)	0.832	76.16 (31.53-194.75)	0.626	7	45 (14-65)	0.333	99.66 (33.97-161.05)	0.632
TT	1	18		69.50		3	30 (28-41)		84.38 (55.11-197.44)	
CC+CT	49	23 (5-94)	0.405	76.16 (23.34-194.75)	0.782	15	36 (14-65)	0.815	89.58 (13.69-161.05)	0.791
**rs4588**										
AA+CA	19	23 (13-94)		80.59 (36.62-183.08)		7	30 (14-46)		84.38 (48.14-197.44)	
CC	31	21 (5-66)	0.477	74.11 (23.34-194.75)	0.412	11	36 (15-65)	0.594	95.56 (13.69-161.05)	0.755
AA	0	-		-		0	-		-	
CA+CC	50	22.5 (5-94)	-	75.13 (23.34-194.75)	-	18	33.5 (14-65)	-	87.89 (13.69-194.44)	-
**rs7041**										
GG+TG	8	21 (9-44)		100.64 (45.01-194.75)		3	26 (23-41)		126.94 (117.75-197.44)	
TT	42	23.5 (5-94)	0.442	72.97 (23.34-161.77)	0.078	15	36 (14-65)	0.487	84.38 (13.69-161.05)	0.048
GG	0	-		-		0	-		-	
TG+TT	50	22.5 (5-94)	-	75.13 (23.34-194.75)	-	18	33.5 (14-65)	-	87.89 (13.69-197.44)	-

^1^ median test.

Caucasian HDs showed no relation between *VDR* SNPs and the analyzed circulating markers. Patients with CT+ CC alleles for A1012G and Cdx2 polymorphism with GA+GG *vs*. AA had significantly higher 25(OH)D levels (*P* = 0.003 and *P* = 0.015, respectively) (Table 7).

**Table 7:** 25(OH)D and DBP levels according to polymorphism distribution in the Caucasian population

		Healthy Donors				Patients				
	25(OH)D (ng/ml)			DBP (μg/ml)		25(OH)D (ng/ml)			DBP (μg/ml)	
	No.	Median value (range)	*P ^1^*	Median value (range)	*P ^1^*	No.	Median value (range)	*P ^1^*	Median value (range)	*P ^1^*
**A1012G**										
TT+CT	45	28.15 (11.26-52.92)		219.62 (101.16-738.06)		31	25.94 (11.00-53.22)		227.98 (79.41-395.70)	
CC	5	29.76 (18.29-60.70)	0.698	203.41 (96.32-304.20)	0.654	4	34.93 (32.25-42.70)	0.067	247.51 (154.17-295.76)	0.894
TT	20	30.11 (11.26-50.63)		201.02 (101.16-738.06)		12	20.72 (11.00-38.22)		227.98 (97.87-385.45)	
CT+CC	30	28.11 (12.36-60.70)	0.586	226.04 (96.32-485.15)	0.806	23	31.73 (19.36-53.22)	0.003	229.13 (79.41-395.70)	0.957
**Cdx2**										
AA+GA	24	28.99 (12.36-50.63)		229.50 (101.16-738.06)		15	25.28 (11.00-47.76)		232.20 (92.33-385.45)	
GG	26	27.80 (11.26-60.70)	0.900	213.20 (96.32-485.15)	0.825	20	30.69 (17.66-53.22)	0.130	221.09 (79.41-395.70)	0.705
AA	4	34.06 (21.86-50.63)		259.66 (130.99-369.87)		4	13.37 (11.00-25.28)		210.09 (97.87-326.03)	
GA+GG	46	28.32 (11.26-60.70)	0.734	219.62 (96.32-738.06)	0.682	31	29.66 (14.25-53.22)	0.015	227.98 (79.41-395.70)	0.653
**Fok1**										
TT+CT	27	28.49 (11.26-43.24)		201.55 (120.85-404.69)		21	29.66 (14.25-53.22)		216.24 (79.41-395.70)	
CC	23	28.15 (12.36-60.70)	0.984	230.28 (96.32-738.06)	0.335	14	23.54 (11.00-52.15)	0.112	233.89 (97.87-338.29)	0.768
TT	8	28.49 (26.76-37.22)		207.33 (133.62-256.55)		5	38.22 (14.25-47.76)		223.76 (166.68-266.59)	
CC+CT	42	28.32 (11.26-60.70)	0.761	226.04 (96.32-738.06)	0.603	30	26.87 (11.00-53.22)	0.442	232.20 (79.41-395.70)	0.736
**rs4588**										
AA+CA	20	27.84 (11.26-60.70)		193.47 (120.85-267.43)		17	25.05 (11.00-38.77)		214.90 (92.33-326.03)	
CC	30	28.78 (12.36-52.92)	0.929	251.82 (96.32-738.06)	0.010	18	32.31 (12.23-53.22)	0.022	270.88 (79.41-395.70)	0.134
AA	1	11.26		142.45		2	16.94 (14.52-19.36)		226.51 (127.00-326.03)	
CA+CC	49	28.49 (12.36-60.70)	0.096	226.04 (96.32-738.06)	0.366	33	28.96 (11.00-53.22)	0.079	227.98 (79.41-395.70)	1.000
**rs7041**										
GG+TG	41	28.15 (12.36-60.70)		236.46 (133.62-738.06)		28	29.31 (12.23-53.22)		257.40 (154.17-395.70)	
TT	9	28.91 (11.26-38.33)	0.631	127.85 (96.32-149.02)	<0.0001	7	24.58 (11.00-38.77)	0.339	100.79 (79.41-198.41)	0.001
GG	16	29.13 (20.69-52.92)		299.34 (195.77-738.06)		13	32.44 (14.52-53.22)		295.76 (206.71-395.70)	
TG+TT	34	27.49 (11.26-60.70)	0.499	182.30 (96.32-278.43)	<0.0001	22	26.01 (11.00-47.76)	0.186	198.41 (79.41-294.16)	0.0003

^1^ median test.

#### DBP SNPs

African HDs with a G allele in the SNP rs7041 of DBP showed higher levels of DBP – not of 25(OH)D – than those with TT homozygosity. Such difference follows the same trend, reaching statistical significance in BCPs (*P* = 0.048) (Table 6). Both Caucasian HDs and BCPs showed about twofold higher levels of DBP with a GG+ TC allele in rs7041. All the comparisons reached statistical significance (Table 7). The presence of CC in rs4588 in HDs and BCPs was related to significantly higher serum levels of DBP (*P* = 0.010) in the Caucasian population (Table 7).

### Association of vitamin D and DBP levels with osteoporosis risk factors

We analyzed serum protein level changes according to osteoporosis risk factors, such as menopausal status, smoking, previous fracture, familiarity, cortisone administration and BMI (Table 8 and 9). Vitamin D levels decreased in Caucasian BC patients with high BMI (*P* = 0.035). Caucasians HD with previous fractures showed lower DBP levels (*P* = 0.048).

**Table 8:** Median values of 25(OH)D levels according to osteoporosis risk factors

	Africans		Caucasians	
	Healthy	Patients	Healthy	Patients
	25(OH)D (ng/ml)Median value (range)			
**Menopausal status**				
Premenopausal	22 (5-94)	31 (14-65)	28.32 (11.26-60.70)	31.49 (25.94-40.48)
Postmenopausal	27 (21-66)	36 (15-48)	27.39 (12.36-34.79)	24.81 (11.00-52.15)
**Smoking habits**				
No	22.5 (5-94)	33.5 (14-65)	28.70 (14.34-52.92)	28.96 (12.23-53.22)
Yes	-	-	24.98 (11.26-60.70)	25.66 (11.00-38.22)
**Fractures**				
No	22.5 (5-94)	33.5 (14-65)	28.0 7(12.36-60.70)	26.74 (12.23-40.48)
Yes	-	-	28.97 (11.26-43.24)	23.91 (11.00-52.15)
**Familiar predisposition**				
No	22.5 (5-94)	33.5 (14-65)	28.91 (11.26-60.70)	26.34 (11.00-52.15)
Yes	-	-	26.76 (12.36-41.47)	25.05 (-)
**Corticosteroid use**				
No	22.5 (5-94)	33.5 (14-65)	28.49 (11.26-60.70)	25.61 (11.00-52.15)
Yes	-	-	-	29.93 (22.78-37.42)
**BMI**				
<20	-	-	29.18 (12.36-50.63)	28.96 (14.25-47.76)
20-25	23 (5-94)	38.5 (14-65)	28.32 (11.26-60.70)	29.93 (21.39-52.15)
>25	11 (-)	21 (19-23)	27.13 (18.29-41.47)	20.06 (11.00-42.70)

All p were > 0.05 except for the comparison of 25(OH)D differences according to BMI in Caucasian patients.

**Table 9:** Median values DBP levels according to osteoporosis risk factors

	Africans		Caucasians	
	Healthy	Patients	Healthy	Patients
	DBP_(μg/ml)Median value (range)			
**Menopausal status**				
Premenopausal	75.13 (23.34-194.75)	84.38 (13.69-197.44)	219.62 (96.32-738.06)	265.25 (103.72-395.70)
Postmenopausal	78.59 (53.58-119.45)	117.75 (33.97-161.05)	211.48 (127.85-299.34)	208.05 (79.41-385.45)
**Smoking habits**				
No	75.13 (23.34-194.75)	87.89 (13.69-197.44)	226.04 (96.32-738.06)	226.64 (92.33-395.70)
Yes	-	-	193.47 (122.53-404.69)	236.65 (79.41-295.76)
**Fractures**				
No	75.13 (23.34-194.75)	87.89 (13.69-197.44)	236.46 (96.32-738.06)	242.57 (79.41-395.70)
Yes	-	-	179.03 (127.85-231.07)	202.56 (92.33-385.45)
**Familiarity**				
No	75.13 (23.34-194.75)	87.89 (13.69-197.44)	213.20 (96.32-738.06)	223.76 (79.41-395.70)
Yes	-	-	232.98 (101.16-293.92)	208.72 (-)
**Cortisone**				
No	75.13 (23.34-194.75)	87.89 (13.69-197.44)	226.04 (96.32-738.06)	208.72 (79.41-395.70)
Yes	-	-	-	290.52 (92.33-295.76)
**BMI**				
<20	-	-	186.26 (122.53-337.03)	223.98 (79.41-395.70)
20-25	74.11 (23.34-194.75)	85.29 (13.69-197.44)	230.30 (96.32-738.06)	235.59 (154.17-338.29)
>25	76.81 (-)	111.25 (95.56-126.94)	236.46 (175.76-299.34)	186.08 (97.87-385.45)

All p were > 0.05 except for the comparison of DBP differences according to the presence of fractures in Caucasian HDs.

**Table 10:** Characteristics of BCPs and HDs in the African and Caucasian populations

	Africans		Caucasians	
	Healthy (*n* = 50)	Patients (*n* = 18)	Healthy (*n* = 50)	Patients (*n* = 35)
	No. (%)	No. (%)	No. (%)	No. (%)
**Median age, years** (range)	35 (25-67)	39 (28-57)	38 (18-66)	58 (39-80)
**Menopausal status**				
Premenopausal	*46 (92.0)*	*13 (72.2)*	*44 (88.0)*	*6 (20.0)*
Postmenopausal	*4 (8.0)*	*5 (27.8)*	*6 (12.0)*	*24 (80.0)*
Unknown/missing	-	-	-	*5*
**Smoking habits**				
No	50 (100)	18 (100)	38 (76.0)	27 (77.1)
Yes	0	0	12 (24.0)	8 (22.9)
**Fractures**				
No	50 (100)	18 (100)	39 (79.6)	15 (51.7)
Yes	0	0	10 (20.4)	14 (48.3)
Unknown/missing	-	-	1	6
**Familiarity**				
No	50 (100)	18 (100)	41 (83.7)	28 (96.5)
Yes	0	0	8 (16.3)	1 (3.5)
Unknown/missing	-	-	1	6
**Cortisone**				
No	50 (100)	18 (100)	49 (100)	26 (89.7)
Yes	0	0	0	3 (10.3)
Unknown/missing	-	-	1	6
**BMI^a^**				
<20	0	0	10 (20.4)	9 (31.0)
20-25	49 (98.0)	16 (88.9)	32 (65.3)	11 (30.0)
>25	1 (2.0)	2 (11.1)	7 (14.3)	9 (31.0)
Unknown/missing	-	-	1	6

^a^BMI, body mass index.

**Table 11:** Characteristics of BCPs in the African and Caucasian populations

	Patients	
	African (*n* = 18)	Caucasian (*n* = 35)
	No. (%)	No. (%)
**Stage**		
I	0	16 (45.7)
II	0	12 (34.3)
III	3 (16.7)	1 (2.9)
IV	2 (11.1)	0
Unknown/missing	13 (72.2)	6 (17.1)
**PgR**		
Negative (<10%)	-	10 (33.3)
Positive (≥10%)	-	20 (66.7)
Unknown/missing	18	5
**HER2**		
Negative	-	27 (90.0)
Positive	-	3 (10.0)
Unknown/missing	18	5
**Mib1**		
Negative (≤20%)	-	20 (66.7)
Positive (>20%)	-	10 (33.3)
Unknown/missing	18	5
**Neoadjuvant chemotherapy**		
No	-	29 (96.7)
Yes	-	1 (3.3)
Unknown/missing	18	5
**Surgery**		
No	0	1 (3.3)
Yes	1 (5.6)	29 (96.7)
Unknown/missing	17	5
**Hormone therapy**		
Adjuvant	0	25 (83.3)
Advanced	12 (66.7)	3 (10.0)
Unknown/missing	-	5
**Chemotherapy**		
Adjuvant	0	11 (36.7)
Advanced	17 (94.4)	2 (6.7)
Unknown/missing	-	5
**Radiotherapy**		
Adjuvant	0	23 (76.7)
Advanced	2 (11.1)	0
Unknown/missing	-	5

Caucasians: BCPs vs. HDs: 25(OH)D: *P*^a^=0.399 DBP: *P*^a^=0.5490.

## DISCUSSION

We performed a study comparing the vitamin D pathway status in a native black Tanzanian and a Caucasian Italian population in an HD and BCP series. We studied 25(OH)D and DBP levels, together with a number of polymorphisms of *VDR* and *DBP* for better understanding the degree of activation of the vitamin D pathway in both populations.

Powe published data from a large case series regarding black and white Americans, reporting DBP levels twofold lower in Blacks than in Whites; our data showed a threefold change [29]. This discrepancy could be due to the differences between African Americans and our Tanzanian population in the case series size, median age (as individuals in Powe's case series were older), gender (as not all individuals were women), provenance and daily habits, such as diet and smoking.

Powe reported that lower DBP levels could explain the coexistence in the black population of low levels of vitamin D and the absence of any skeletal problems. This theory is in line with the previously reported hypothesized role of DBP as regulator of vitamin D [18, 19], by which low levels of DBP can counterbalance low levels of vitamin D. Some authors recently reported that besides entering the cells as an unbound hormone, vitamin D can enter cells bound to DBP through an endocytosis uptake mechanism [36–37]. Further studies are needed to understand whether this new discovery impacts the role of DBP in vitamin D regulation.

The DBP polymorphisms rs7041 and rs4588 analyzed by Powe and in the present study were related to racial differences in circulating DBP levels [29]. In particular, T allele was more frequent and A allele was less frequent in Blacks at rs7041 and rs4588, respectively. These variants were related to lower and higher DBP levels [29], respectively. Our data showed a higher prevalence among Blacks of rs7041 polymorphism in the *DBP* gene associated with low levels of DBP, potentially resulting in bioavailable 25(OH)D levels similar to those in Whites, despite their lower levels of total 25(OH)D.

Unlike Powe et al., we analyzed a number of SNPs related to *VDR*, such as A1012G, Cdx2, and Fok1. The first and the second are located in the promoter regions of the *VDR*, which could influence *VDR* expression [38–39].

G allele of Cdx2 decreases *VDR* gene transcription. Fok1 polymorphism, instead, produces two alleles distinguished by the presence or absence of the Fok1 restriction site. TT allele encodes full-length VDR protein, while CC allele encodes a shorter protein. Our data showed that the Tanzanian population was more likely to have Fok1 CC homozygosity, which resulted in a VDR protein with higher transcriptional activity, and Cdx2 AA homozygosity, related to an increase in the transcription of *VDR* gene. Considering the differences in allelic variant distribution between cases and controls within the African population, we interestingly observed that BCPs and HDs have 38% and 70% of CC for Fok1 SNP, respectively. Given that vitamin D could act as a shield against BC development [10–15] since CC is related to a higher activity of VDR, this datum suggests that the vitamin D pathway status can play a protective role against BC development in Africans.

A number of papers, including several meta analyses reporting data on thousands of individuals, evaluated the role of Fok1 and Cdx2 and other *VDR* polymorphisms in BC risk increase with mixed results [40–50]. Our series is still overly limited to allow for a BC risk analysis.

Our paper has few limitations: first, the small case series size impeded any statistical adjustment according to population characteristics and time of the year of sample collection. In fact, Caucasians were all enrolled in the same period, whereas Tanzania's lower latitude and consistent sunlight exposure made the time of collection less relevant. Secondly, HDs and BCPs of the two populations were not matched by age: African BCPs were younger than Caucasians BCPs, as Africans developed BC earlier than Caucasians [27, 51].

Our results demonstrated that the two analyzed populations are different not only with respect to DBP levels, but also to the allelic variant distribution of *VDR* and *DBP* SNPs. According to our results, Africans have innate higher levels and stronger activity of VDR, with a consequent higher degree of activation of the vitamin D pathway. These alterations, alongside those in DBP levels previously reported in literature, may be responsible for the observed racial differences in the total vitamin D levels in absence of symptomatic manifestations of vitamin D deficiency in Blacks [29]. So far a lower BC incidence has been observed in Africans and African Americans than in white Americans, even if BC incidence in African Americans is currently rising [27, 52]. Although these differences also stem from several genetic and environmental factors, we hypothesize that one of the mechanisms involved is that Africans, despite vitamin D deficiency, can be protected from BC thanks to a higher activity of the vitamin D pathway.

Although the hypothesized mechanism awaits to be confirmed, it nevertheless elicits further investigation on genetic racial differences with respect to cancer.

## MATERIALS AND METHODS

### Study design

This prospective study was carried out at the Istituto Scientifico Romagnolo per lo Studio e la Cura dei Tumori (IRST) IRCCS (Meldola, Italy) in collaboration with the Bugando Medical Centre (Mwanza, Tanzania). Our primary objective was to compare 25(OH)D levels in an African and a Caucasian population of HDs and BCPs. We aimed to observe differences between the populations, and between cases and controls. We investigated also other markers of the vitamin D pathway, such as DBP levels and 5 polymorphisms (SNPs) of *VDR* (FokI, Cdx2 and A1012G) and *DBP* (rs4588 and rs7041), correlating them with the vitamin D trend and the BC biological features of the African and Caucasian BCPs. The protocol was reviewed and approved by the local ethics committee and performed according to the Good Clinical Practice and the Declaration of Helsinki. The patients gave their written informed consent to take part in the study.

### Case series

Peripheral venous blood (PB) samples were obtained from 85 Caucasian (50 HDs and 35 BCPs) and 68 African women (50 HDs and 18 BCPs). HDs and BCPs were enrolled between 2014 and 2015. HDs and BCPs were eligible if not taking supplementation of vitamin D. Case series details are reported in Tables 10 and 11.

### Biological samples

For each HD and BCP 2 tubes of PB were collected: one 5 ml tube without anticoagulant and one 3 ml tube with EDTA. The first tube was left to coagulate for 30 minutes at room temperature and centrifuged at 2,000 *g* for 15 minutes, after which serum was stored at -80°C until assays were performed. Blood from the second tube was aliquoted and stored at -20°C until DNA extraction. Each blood and serum sample was also stored in Whatman filter paper (Milan, Italy) for dried blood spot (DBS) and dried serum spot (DSS) sampling. Briefly, 50 μl of serum/entire blood were spotted in 5 consecutive filters of a Whatman paper card allowed to dry overnight and stored in a plastic bag with desiccant at -20°C until use.

### 25(OH)D levels

Serum 25(OH)D level is currently the best representative form of vitamin D for measuring vitamin D status. For the Caucasian population levels of 25(OH)D were quantified in serum by an IVD commercial immunoenzymatic assay by Biovendor (Hamburg, Germany) following the manufacturer's instructions. For the African population, 25(OH)D levels were quantified by serum spotted in filters by ZRT Laboratory using liquid chromatography/tandem mass spectrometry (LC-MS/MS), which is also used by the CDC Nutrition Laboratory to obtain accurate 25(OH)D values [53]. Before merging the vitamin D levels obtained with the 2 methods, we tested the reproducibility of the 2 assays analyzing 13 samples with both methods. Results were considered satisfactory as the coefficient variation was < 15% in all the comparisons. The 25(OH)D levels observed included free and bound vitamin D.

### DBP levels

DBP levels were evaluated by a commercial immunoenzymatic kit (R&D system) starting from DSS stored as described above. One spot for each sample was allowed to thaw, cut in small pieces and put into a 1.5 ml tube with 400 μl of PBS. Samples were shaken overnight at 4°C [54]. The protein eluate obtained was utilized as a serum sample for DBP detection according to the manufacturer's instructions, after appropriate dilutions.

### Genotyping

Genomic DNA from the Caucasian population was extracted from PB samples by QIAamp DNA Mini Kit (Qiagen, Hilden, Germany) according to the Blood and Body Fluid Spin protocol. Genomic DNA from the African population was instead extracted by QIAmp DNA micro kit according to the Dried Blood Spot protocol. DNA quantity and quality were assessed by Nanodrop 1000 (Celbio, Milan, Italy). Fifteen ng of DNA were used to detect each SNPs by real-time PCR using TaqMan SNP assays and Taqman genotyping Master mix ThermoFisher Scientific in a total volume of 10 μl according to the manufacturer's instructions. PCR reaction consisted in: 10 minutes at 95°C, 15 seconds at 95°C for DNA denaturation, and 1 minute at 60°C for annealing and extension for 40 cycles.

The real-time PCR profiles of homo- and hetero-zygosis of Rs7041 were not clear. For this reason 10 additional samples were analyzed only for Rs7041 by direct sequencing to confirm real-time PCR (primer sequences: F: TCGAAGAGGCATGTTTCACT; R:GCAGTTGGA GGCAAAGTCTG).

After sequencing and genotyping, the Hardy-Weinberg equilibrium was determined by the Haploview program (Version 4.2) (Broad Institute of MIT and Harvard University).

### Statistical analyses

Descriptive analyses were presented for demographic and clinical characteristics. Categorical variables were presented as numbers and percentages, and continuous data as median and range. The distribution of serum vitamin D levels was categorized as deficient (< 20 ng/mL), suboptimal (20 to 29 ng/mL), optimal (30.50 ng/mL), upper-normal and above (> 50 ng/mL). The DBP levels were categorized according to the tertile of distribution. Differences between populations, between vitamin-D-deficient and non-vitamin-D-deficient women with respect to the demographic and clinical characteristics, and between allelic frequency of SNP in our Caucasian and African case series and in a 100-genome dataset were examined with the Chi-square test. The Spearman's correlation was used to assess the correlations between vitamin D and DBP levels considered as continuous variables. The nonparametric median test was used to estimate the correlation between the continuous variables in the two populations (African or Caucasian) or subject status (HD or BCP). All statistical analyses were conducted with SAS Statistical software, version 9.4 (SAS Institute).
